# The effect of body composition on strength and power in male and female students

**DOI:** 10.1186/s13102-021-00376-z

**Published:** 2021-11-28

**Authors:** Ghassen Ben Mansour, Asma Kacem, Mohamed Ishak, Laurent Grélot, Foued Ftaiti

**Affiliations:** 1 Department of Physiology and Functional Explorations, IBN EL JAZZAR Medicine Faculty, Sousse, Tunisia; 2 Sports Training Department, Dubai Police Academy, Dubai, UAE; 3 Institute of Sport and Physical Education, Central University, Sfax, Tunisia; 4 Institute of Sport and Physical Education, Manouba University, Ksar-Said, Tunisia; 5 Aix-Marseille University Institute of Technology, dept HSE, 13708 La Ciotat & HIA Laveran, 13013 Marseille, France

**Keywords:** Sex difference, Fat mass, Ballast, Power, Strength

## Abstract

**Purpose:**

The aim of this study is to determine and to compare the effect of sex differences in percentage of body fat on the strength and power performances of the legs and arms during short maximal exercise.

**Methods:**

72 male and 64 female students aged 20 to 23 years were enrolled in this study. After assessing their morphological characteristics (body mass, height and percentage of fat mass), a squat jump test (SJ), a 5 successive jump test (5JT), a hand gripping (HG) and back strength (BS) tests have been conducted for each subject. Male students were re-tested after being weighed down with a weight equivalent to the mean differences in body fat recorded between the two sexes in the form of a loaded worn vest.

**Results:**

Male are 15.7% heavier and 7.4% taller and presented a percentage of fat mass (17.2 ± 1.8%) significantly (*p* < 0.001) lower than that of women subject (25.0 ± 2.5%) (difference male vs female for fat mass: -45.5%). HG, BS, 5JT and SJ performances were significantly higher in males (44 ± 5 kg, 141 ± 2 kg, 11 ± 1 m and 32.4 ± 2,7 cm, respectively) than in females (31.0 ± 4 kg, 81.6 ± 13 kg, 8.7 ± 0.7 m and 21.1 ± 1.9 cm, respectively). In the control (unloaded) condition, the relative difference between males and females represented 23.5% and 34.7% of the male performances for 5JT and SJ, respectively. In the weighted condition, the relative difference between weighted males and females still represented 11.7% and 23.8% of the weighted male performances for 5JT and SJ, respectively. Cancelling the sex difference in fat mass by adding weight in males reduced by 50.1% the sex difference during 5JT and 31.4% and 71.7% for hight and power results, respectively during SJ test.

**Conclusion:**

During short and maximal exercise, male performed better with their hands, back and legs than female students. Excess fat for female students has a disadvantageous effect on vertical and horizontal jumps performances. The persistence of sex differences after weighting of male students indicates that body fat is responsible for 30 to 70% of the observed differences between sexes performances and power outcomes during jump tests.

## Introduction

Both age and gender are strongly related to physical performance throughout childhood and adolescence [1]. Gender differences in motor skills seem to be explained by interactions between environmental and biological factors [2]. According to Podstawski et al. [3] biological variables can explain only 30% on average. These gender-related differences in anthropometric characteristics and motor skills are noticed at all stages of life [3].

Gender is a major determinant of the best athletic performances, due to various morphological and physiological differences [4]. Women generally cannot perform at the same level as men during tasks requiring high levels of strength, muscular endurance, or physical work capacity [5].

The overall body composition characterizes the size and configuration of the body, which is often described by anthropometric measures such as body mass, skin fold thickness, circumferences to assess hip/thigh ratio and body mass index [6]. These anthropometric properties of the athletes are an essential prerequisite for a successful presence in the same sport, affecting the athlete's performance, and are necessary to obtain excellent sports performances [7]. Anthropometric changes associated with growth and maturation are important factors affecting motor strength and performance [8].

According to the action of sexual hormones, a progressive increase in body fat was observed in adolescent girls with sexual maturation [9]. The percentage of body fat is the amount of fat stored in the body and does not account for lean body mass and muscle mass.

Sex differences are based on an increase in total body fat mass, and more specifically in the lower limbs during puberty in girls, while boys had increased lean body mass [10]. Boys become progressively longer in skeletal length and muscle width and size, compared to girls [11].

The muscular volume being much lower in women because of the lower production of testosterone, and the percentage of fat mass due to the influence of estrogen being higher, the female performance can never match those of their male counterparts [12].

Sex differences in the efficacy/efficiency of exercise to change body composition (i.e. fat loss) may be mediated by differences in hormonal responses [13].

Sex-based differences in the hormonal response may be manifested in changes to appetite, energy intake, and energy expenditure that more effectively stimulate appetite and energy intake (and possibly suppress energy expenditure) in women than in men.

According to Podstawski et al. [3] strength is directly related to the number and dimensions of muscle fibers (i.e. diameter of type I vs. type II) recruited and the frequency of activation by the central command. Muscle strength improves with age in mid-childhood and adolescence, but the pattern of improvement is influenced by many factors such as sex, body size, maturity, and to some extent motor skills and physical activity [14]. In the general population absolute strength level is generally 40℅ stronger in men than in women [15]. At the same age, absolute maximal power is always higher in boys than in girls, and the difference increases after puberty [16]. This is in agreement with Doré conclusions [10] which confirms a girl-boy differentiation from the beginning of puberty. According to this author, girls have lower maximum power values than boys, even when values are expressed as a function of body size.

The importance of the differences in strength between sexes depends on the muscle groups studied. At the level of the lower limbs, the level of strength of women corresponds to 60–80% of that developed by the man against 60% at the level of the upper limbs [17]. This difference is due to the number of muscle fibers recruited as well as the muscle size in boys. In addition, the female musculature may contain more intramuscular fat and connective tissue [17] than in humans. The variations in strength and power between man and woman appear for the lower limbs as for the upper limbs when the performances are expressed in absolute values [18]. When expressed relative to body mass or to lean mass, these differences cancel out for the lower limbs while they persist for the upper limbs [19].

Miller et al. [17] indicates that sex-related strength differences are more pronounced at the top. Therefore, the difference in grip strength could at least partly be attributed to the fact that women tend to have a lower portion of their lean mass located in the upper body. These sex differences can be attenuated or accentuated according to the cultures and habits of each country (i.e. sport participation by youth in both school and clubs). In Tunisia and despite the equality between women and men and the development of women's sport, some gray areas are to be mentioned in view of the number of women engaged in civilian sports and the achievement of sports performances at national level and international. For example, handball where only 28.85% of the licensees are female.

The aim of the present study was to determine and compare the morphological characteristics of two groups of adults of different sexes and to verify the effect of sex differences in percentage of body fat on the strength and power performances of the legs and arms during short maximal exercise.

On the basis of the literature data, we reached the following assumptions:

1. Young adult female would be smaller and weigh less than their male counterparts.
2. The difference in body composition (i.e. both the muscle mass and the percentage of fat) is responsible for the differences in strength and power performances observed between the two sexes.

Hence, we aimed to quantify the strength and power differences of the lower and upper limbs between male and female students prior and after having loaded males with additional weights to compensate the natural difference in fat mass between the two sex groups.

## Methods

### Subjects

We enrolled a total of 72 male and 64 female Tunisian students aged 21 ± 2 and 22 ± 3 years old, respectively.

The inclusion criteria were: (a) healthy student volunteers who practiced similar volume of physical activity as part of their university physical weekly training, i.e. 5 h per week; (b) male subjects exhibiting a % body fat around 17%; (c) Female subjects with a % body fat around 25%. The exclusion criterions were (a) participation in additional physical activity (i.e. part of training in civilian clubs); (b) smokers and subjects how take any medication or nutritional supplements.

All women were tested in the middle of the follicular phase (PF) of the menstrual cycle between days 4 and 8 after menstruation.

The Study has been approved by the local ethics committee of the Medicine Faculty of Sousse in Tunisia and has therefore been performed in accordance with the ethical standards laid down in the 1964 Declaration of Helsinki (Revised in 2013).

All participants gave their informed formal consent prior to their inclusion in the study and that details that might disclose the identity of the subjects under study have been omitted.

### Anthropometric parameters

#### Body mass

Body mass was measured using a Tanita balance (model TBF-300). The subject must stand upright without assistance. It must stand still in the center of the weighing pan, the body weight evenly distributed over the two feet slightly apart. Shoes and clothing should be removed, except for underwear that can be kept. Body mass was recorded in kilograms ± 100 g.

#### Height

Subject height was measured using a measuring rod (graduated in centimeters; a standard anthropometric kit; Harpenden type, Switzerland) comprising a horizontal cursor which is brought into contact with the highest point of the head. The subject must be barefoot, and little dressed so that the experimenter can observe the positioning of his body as well as his posture. He should stand as straight as possible on a flat surface, the weight distributed evenly on both feet, heels joined, and the head placed so that the line of sight is perpendicular to the body. The arms hang freely along the body, and the head, back, buttocks and heels are in contact with the flat, vertical surface behind the subject. Then, subject took a deep breath, and the measurement was made just before expiration. The movable cursor is brought into contact with the highest point of the head, pressing it down enough to compress the hair. Height was measured in meters ± 0.1 cm.

#### Body mass index

Body mass index (BMI in kg/m^2^) was calculated for each subject by dividing body mass (in kg) by the square of the height (in m).

#### Body fat

Skin pliers (Harpenden type, Switzerland) was used to measure skin folds. These measurements have always been carried out by the same experimenter, perfectly trained in this technique. Skin fold measurements were taken at 4 traditional sites (i.e. biceps: front side middle upper arm, triceps: back side middle upper arm, subscapular: under the lowest point of the shoulder blade, supra-iliac: above the upper bone of the hip) on the dominant side of the body. The measurements were carried out three times by the same person according to the technic established by the international biological program [20]. The average of the three measurements determined the value for the measured skin fold. The percentage of body fat was estimated from the skin folds using the equation of Durnin and Womersely [21] for young adults aged from 20 to 29 years.

$$\% \,{\text{body}}\,{\text{fat}} = \left( {{4}.{95}/\left[ {{\text{Density}} - {4}.{5}} \right]} \right)*{1}00$$

where D = 1.1631 − (0.0632 * Log sum of the 4 folds) for male subjects; D = 1.1599 − (0.0717 * Log sum of the 4 folds) for female subjects.

### Weighting protocol

Male students were weighted by wearing a loaded worn vest (CAPITAL SPORTS Monstervest). This vest features weight separately removable 30 kg metal weights and a soft padding all around and a adjustable nylon strap with Velcro.

The ballasted weight (kg) was calculated as follows:

1. ∆ Female/Male (%) body fat = % Female mean body fat − %Male mean body fat
2. Ballast weight (kg) for each male subject = weight subject (kg) × ∆ Female/Male (%) body fat
3. The calculated ballast weight (kg) was entered using 1, 0.5 and 0.1 kg weights introduced into the jacket pockets.

### Physical parameters

#### Hand strength test

The handgrip (HG) force can be quantified by measuring the static force that the hand can exert around on a hand-held manual dynamometer (Takei Physical Fitness Test). The test protocol consisted of three maximal voluntary isometric contractions. Instructions and demonstrations were given to the participants according to the standard recommendations [22]. The subjects were seated, elbows bent 90° and supported at the time of the measurement [23]. We asked the subject to grasp and squeeze the dynamometer with the dominant hand with maximum force and hold it for at least 3 s. We collected three measurements of each subject and the best was maintained. The handle force is recorded in kilograms of maximal force. A rest interval of thirty seconds was scheduled between each measurement.

#### Back strength test

Back strength (BS) was measured in kilograms (kg) using a back and leg dynamometer (type Takei Physical fitness test), previously described by Koley et al. [24, 25]. The subjects stood on the foot of the dynamometer, feet shoulder-width apart, and grabbed the handlebars positioned on the thigh. The length of the dynamometer chain has been adjusted so that the legs are straight, and the back is bent at an angle of 30° to position the bar at the kneecap. The subjects were then asked to straighten their backs (i.e. stand up) by bending their knees and lifting the dynamometer chain, applying the pulling force exerted on the handle, pulling as hard as possible up. Subjects completed three trials, with the highest score recorded in kilograms as a measure of maximum back strength under isometric conditions. A rest interval of thirty seconds was scheduled between each test.

#### Five jumps test (5JT)

The 5 JT is a test reflecting the explosive force of the lower limbs. He was carried out in a covered sports hall, with a floor covered with wooden parquet. The distance in meters made by the subject during the five jumps was measured using a double decameter. The average performance per hop was obtained by dividing the total length of the 5JT by five (in m). From the right station, legs spread shoulder-width apart, the subject performs five leaping strides. He jumped on one leg (right or left) raising the knee and arms in front (front lunge). During the fifth stride, the subject brought the two legs together to arrive at the same starting position. This test requiring good motor coordination and a learning-training session took place one week before the evaluation. Subjects completed three trials during the assessment session. The best performance was recorded and expressed in meter. All tests were accompanied by verbal encouragement to stimulate performance and in a similar manner for all subjects [26].

#### Squat jump test (SJ)

The height and power of the vertical jump were evaluated using an optical system (Optojump, Microgate, Italy). During the SJ, the subject was asked to remain crouched for three seconds. On the count of three, the subject was asked to jump as high as possible. A successful attempt was a test in which there was no downward movement or counter-movement before the execution of the jump. Height measured during SJ was expressed in cm. For the vertical jump test, participants had to jump vertically for maximum height and land in the same position and in the same place after takeoff to avoid any lateral or horizontal movement [27].

Verbal encouragement was constantly given to ensure high motivation. All subjects carried out 3 tests for each jump and the best of the three attempts was selected. The jumps were separated by 2 min of recovery.

#### Jump tests with ballast

Three days later, male subjects were asked to repeat the same jumps tests (5JT and SJ) by ballasting them. The addition of external weight to the body was achieved by wearing a weighted vest using loads equivalent to the differences in % of fat mass noted between the two sexes. Care was taken to ensure that the added weight was secure, comfortable and did not interfere with the jumping movements.

## Protocol summary

**Table:** 

Gender	Step 1						Step 2				Step 3	
	n֠	Age (years)	Anthropometric measurements				Physical measurements				Weighting protocol	
Male	72	21 ± 2	Body mass	Height	Body mass index	Body fat	Hand Strength Test	Back strength test	Five Jumps Test (5JT)	Squat Jump Test (SJ)	5JT	SJ
Female	64	22 ± 3										

### Statistical analysis

Before ballasting male subjects, the comparison of the morphological and physical parameters recorded in the two sex groups was performed using a one-way (Gender: male and female) analysis of variance ''ANOVA''. After ballasting male subjects, jump tests results were compared using one way analysis of variance (Weighting: weighted male, non-weighted male and non-weighted female). When the analysis showed significant results, a ‘’Post Hoc’’ test comparison "Scheffé Test" was used to determine the importance of the differences between the mean values of the different groups. The significance threshold is conventionally set at *p* < 0.05.

## Results

*Differences between male and female* (∆ F/M%) of anthropometric and physical characteristics are expressed in % of the values of male subjects.

### Morphological parameters

Subjects body mass, height, body mass index and percentage of body fat were presented in Table 1.

**Table 1:** Anthropometric characteristics of the subjects. ∆ F/M (%): difference between female and male subjects expressed as in % of the value of male students

Groups Parameters	Male (n = 72)	Female (n = 64)	∆ F/M (in %)
Body mass (kg)	72.8 ± 7	61.4 ± 7	15.7 *p* < 0.001
Height (m)	1.75 ± 0.05	1.62 ± 0.05	7.4 *p* < 0.001
BMI (kg/m^2^)	23.5 ± 2	23.23 ± 2.01	0.9 NS (*p* < 0.56)
Body fat (%)	17.2 ± 1.8	25.0 ± 2.5	− 45.5 *p* < 0.001

#### Body mass

Male students were 15.66% significantly heavier than female students with a body mass of 72.8 ± 7 kg for men and 61.4 ± 7 kg for women (F = 75.65; *p* < 0.001).

#### Height

The average height of the male and female students were 1.75 ± 0.05 m and 1.62 ± 0.05 m, respectively. Women are 6.9% smaller than men (F = 148.6; *p* < 0.001).

#### Body mass index

Statistical analysis did not reveal any statistical differences between male (23.5 ± 2 kg/m^2^) and female BMI (23.2 ± 2 kg/m^2^).

#### Body fat

Male subjects exhibited a percentage of fat mass (17.2 ± 1.8%) significantly (F = 57.5; *p* < 0.001) lower than that of female subject (25.0 ± 2.5%) (i.e. sex differences: -45.5%).

### Physical parameters

Physical performances of both female and (unloaded) male students during tests were illustrated in Table 2. Table 3 represented the physical performances of the two groups after wearing addition weight to men (i.e. weighted Male group).

**Table 2:** Absolute and relative (to body mass: BM and to lean mass: LM) hand gripping and back strength performances of male and female subjects

Groups Tests	Male (n = 72)	Female (n = 64)	∆ F/M (%)
Hand grip (HG) (kg)	44.0 ± 5	31.0 ± 4	30.8 *p* < 0.001
Hand grip relative to body mass (HG/BM)	0.6 ± 0.08	0.5 ± 0.06	18.03 *p* < 0.001
Hand grip relative to lean mass (HG/LM)	0.74 ± 0.10	0.67 ± 0.06	9.46 *p* < 0.01
Back strength (BS) (kg)	141.0 ± 18	81.6 ± 13	43.7 *p* < 0.001
Back strength relative to body mass (BS/BM)	2 ± 0.26	1.3 ± 0.2	31.1 *p* < 0.001
Back strength relative to lean mass (BS/LM)	2.4 ± 0.3	2 ± 0.2	15.7 *p* < 0.01

Data presentation as in Table 1

**Table 3:** 5 JT and SJ performances of male and female subjects before and after ballasting

Groups Tests	Male	Weighted male	Female	∆ M/F (%) Male vs. Female	∆ F/wM (%) Female vs weighted Male	∆ reduction (%) Male vs. Female (after cancelling the sex difference in fat mass)	∆ wM/M (%) Male vs. Weighted male
5JT (m)	11.4 ± 0.5	9.9 ± 0.5	8.7 ± 0.71	23.5 *p* < 0.001	11,7 *p* < 0.001	50,1	13.3 *p* < 0.001
SJ							
Height (cm)	32.4 ± 2.7	27.8 ± 2.5	21.1 ± 1.9	34.7 *p* < 0.001	23.8 *p* < 0.001	31,4	14.3 *p* < 0.01
Power(Watt/kg)	15.8 ± 1.40	12.8 ± 1	11.9 ± 1.1	24.9 *p* < 0.001	7.0 *p* < 0.05	71,7	19.2 *p* < 0.01

Data presentation as in Table 1.

∆ F/wM (%), difference between female and weighted male students expressed as in % of the value of weighted male students; NB, data obtained in the tests performed on the lower limbs (i.e. 5JT and SJ), are not relative to the body mass or lean mass

#### Manual strength (handgrip test)

Manual strength performance (HG) was 30.8% significantly (F = 169.6; *p* < 0.001) higher in male (44.0 ± 5 kg) compared to female subjects (31.0 ± 4 kg).

When expressed relative to body mass and to lean mass the male–female differences was reduced to 18.0 (*p* < 0.001) and 9.46% (*p* < 0.01), respectively (Table 2).

#### Back strength

The back strength (BS) results showed that the male subject’s performance (141 ± 18 kg) was 43.7% stronger (F = 351.9; *p* < 0.001) than the female subjects (81.6 ± 13.3 kg).

When expressed relative to body mass and to lean mass the male–female differences was reduced to 31.1 (*p* < 0.001) and 15.7% (*p* < 0.01), respectively (Table 2).

#### The 5 Successive Jumps test (5JT)

5JT performances was 23.5% significantly (F = 105; *p* < 0.001) higher in men (11.4 ± 0.5 m) compared to women (8.7 ± 0.7 m) (Table 2). After ballasting the male students, the differences between weighted male and female groups were attenuated to 11.7% but performances remain significantly higher (*p* < 0.001) in weighted male (9.9 ± 0.5 m) than in female group (8.7 ± 0.7 m) (Table 3). This corresponds to reduction of 50.1% of the sex difference.

#### The squat jump (SJ)

The average height recorded in male and female students during SJ was respectively 32.4 ± 2.7 cm and 21.1 ± 1.9 cm. Thus, men's jump performance was 34.7% higher than that of their female counterparts (F = 155.7; *p* < 0.001) (Table 3).

Similarly, power jump performance of the male students (15.8 ± 1.4 Watt/kg) was 24.9℅ significantly higher (F = 75.2; *p* < 0.001) to those of the female subjects (11.9 ± 1.1 Watt/kg) (Table 3).

After ballasting, male student height and power performances during SJ were attenuated to 27.8 ± 2.5 cm and 12.8 ± 1 Watt/kg and sex differences were attenuated to 23.8% (*p* < 0.001) and 07.01% (*p* < 0.05), respectively (Table 3).

## Discussion

The aim of our study was to determine and compare the morphological characteristics of two groups of adults of different sexes and to verify the effect of sex differences in percentage of body fat on the strength and power performances of the legs and arms during short maximal exercise.

Our study reveals that male students are 7.4% (i.e. 13 cm) taller and 15.7% (i.e. 11.4 kg) heavier than female ones. Our results are comparable to those of Shephard [14] reporting that men are 13 cm taller and ≈ 14 to 18 kg heavier than women. In this line, male and female students of our study exhibit a comparable BMI (23.5 ± 2 kg/m^2^ and 23.2 ± 2 kg/m^2^, respectively). This result is in agreement with values reported by Kacem et al. [26] i.e. 23.8 kg/m^2^ and 22.9 kg/m^2^ for men and for women, respectively. We observed also that male students have a significantly lower body fat percentage than female ones (∆ F/M in %: − 45.5%). In the present study, this choice of important differences in percentage of body fat is deliberate in order to analyze the effect of sex differences in percentage of body fat on the strength and power performances. These differences are higher than those reported by Kacem et al. [26] and Shephard [14] with mean differences around 10 to 15%. In the other hand, our results reveal a significantly higher physical strength and power of both limbs in male compared to female students.

The evaluation hand gripping (HG) reflects the span of the hand and the body size [28]. However, international literature suffers from a lack of reference values for this parameter, in particular among female populations. Our results showed that male subjects performed 30.8% better than female subjects (F = 169.6, *p* < 0.001). This is in agreement with results reported by Angst et al. [29], according to which greater grip strength was observed in men compared to women, whatever the age.

According to Grélot (personal communication, 2020) a 39.6% sex difference in 18.5-year-old French students (i.e. male, n = 99, HG = 53.3 ± 11.7 kg vs female, n = 23, HG = 32.2 ± 6.6 kg) was measured.

In this line, Gómez-Campos et al. [30] reported significant differences between the two sexes regardless of their biological age. Sartorio et al. [31] showed that the most important factor influencing the strength of the handle seems to be always sexes, or in particular the sexual hormones. In adolescence, adipose tissue is predominant in girls while muscle mass increases considerably in boys [30]. Lean body mass is linked to sex hormones, which are more common in boys than in girls [31, 32]. Growth and testosterone have more effects on grip strength than in girls [33]. In this line, Leyk et al. [34] have shown that the strength of handful was linearly correlated with lean body mass in a large sample of German adults’ of 1654 men and 533 women Pizzigalli et al. [35] reported that height, arm length and body mass have a positive effect on hand gripping performance.

Miller et al. [17] indicate that the differences in strength linked to sex are more pronounced in the upper part of the body. Therefore, the difference in strength can be attributed to the fact that women have less lean body mass in the upper body [17]. The variations in strength and power between man and woman appear for the lower limbs as for the upper limbs when the performances are expressed in absolute values [18]. When expressed relative to body mass or to lean mass, these differences cancel out for the lower limbs while they persist for the upper limbs Weber et al. [19]. In this line, our results revealed a significant difference between male and female students’ groups for the higher (Table 2) and the lower limbs (Table 3). When expressed relative to body mass and to lean mass, these differences were reduced but persist significantly (Table 2).

Our results reveal significantly (*p* < 0.001) higher performances for back strength exercise in men compared to women subjects. Male students are 44.4% stronger than female ones. These results are in agreement with those reported by Koley et al. [24], in a male student population compared to their female counterparts. In addition, Koley et al. [24] reported that regardless of age, male subjects have higher average back strength values than their female counterparts. According to the same authors, back strength is positively correlated with higher testosterone levels in men. In this line, Podstawski et al. [3] and Seger and Thorstensson [11] have shown that biological maturation has a significant impact on muscle strength during puberty. In addition, during adolescence period, adipose tissue is predominant in girls while muscle mass increases considerably in boys.

Our results showed that 5JT male performance was about 23.5% better than female one (*p* < 0.001). Our results are in agreement with those of Kacem et al. [26] for which man produces greater power during short-term efforts. Maud and Shultz [36] state that the anaerobic power and the anaerobic capacity of men are greater than those of women. However, these observed differences decrease after normalization to body mass and vanish when normalized to lean mass. According to Kacem et al. [26], during 5JT test sex differences persist only at age of 14 years (30.4%, *p* < 0.001) when performance was normalized to the muscle volume of the legs and disappear at the age of adult (2.1%). Mayhew and Salm [37] suggest that anaerobic power in both sexes is related to anthropometric dimensions and the muscular strength that results from it. These same authors reported that body size and strength are the major factors explaining sex differences of the power of the lower limbs. According to Wells [38], the hormonal differentiation observed at puberty causes a substantial increase in body fat for female subjects and in muscle mass for male subjects. Similarly, Doré et al. [10] suggests that gender differences were due to the increase in total fat mass, and more specifically to the increase in lower extremity fat during puberty in girls, while boys had increased lean body mass. Kacem et al. [26] have shown that the percentage of body fat represents a factor, which disadvantages performance during brief and intense efforts (i.e. 5JT) in both young and adult women.

Since the percentage of body fat is a physical characteristic and since women generally have values of the order of 10% higher than in men, they often remain at a disadvantage [36] because they have to lift or support more unnecessary mass during jumping and racing efforts.

**Fig. 1:**
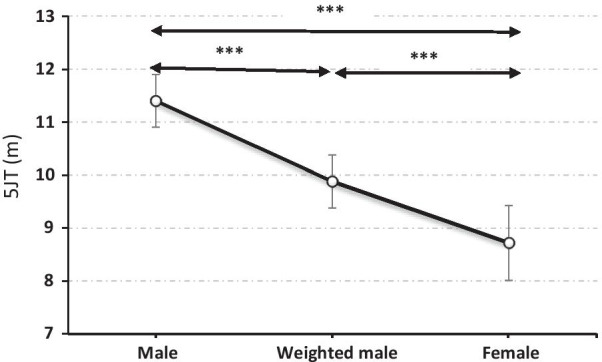
5JT performance for male (male: before ballasting; weighted male: after ballasting) and female students. ***p < 0.001

When ballasted, males’ performances in 5JT (11.4 ± 0.5 m) decreased by 11,8% but remained significantly (*p* < 0.001) higher than female performances. This corresponds to a significant (*p* < 0.001) reduction of 50.1% of the male vs female differences (Fig. 1).

The vertical jump (i.e. the normalized SJ) is commonly used as an index of the power of the lower limb [39]. The vertical jump performance is an important element for a successful performance in several sports. In this study, the results of the SJ revealed a significantly higher performance (+ 34.7%, *p* < 0.001) in male students compared to female ones.

Similarly, the power outcome during SJ of male students was 24.9% significantly higher (*p* < 0.001) to that of the female ones. Our results are in agreement with those reported by Abidin and Adam [40] and Hanjabam et al. [41].

According to these authors, the difference in jump performance between the two sexes is linked to the higher body fat mass in women. Even for confirmed athletes Abidin and Adam [40] reported that female have a higher percentage of body fat than male, in particular due to that stored in the hips and chest. Therefore, male athletes have the advantage to jump higher [42].

**Fig. 2:**
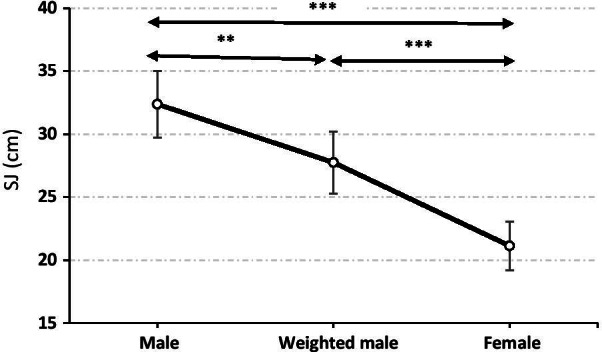
Squat jump height performance for male (weighted male: after ballasting) and female students. **p < 0.01; ***p < 0.001

**Fig. 3:**
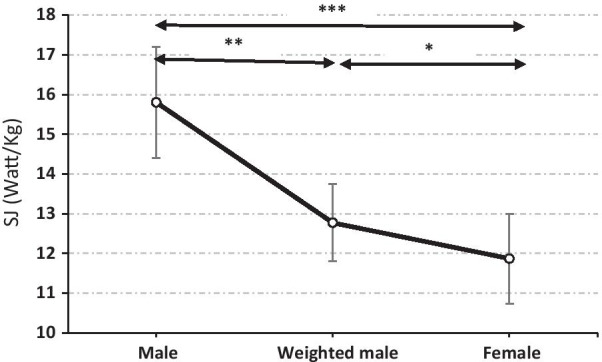
Squat jump power performance for male (weighted male: after ballasting) and female students. *p < 0.05; **p < 0.01; ***p < 0.001

When ballasted, male performances in SJ performances (27.8 ± 2.5 cm) decreased by 10.8% but remained significantly (*p* < 0.001) higher than female performances (21.1 ± 2 cm). The relative difference between weighted males and females still represented 23.8% (*p* < 0.001) and 7.0% (*p* < 0.05) of the weighted male heigh and power performances, respectively. Cancelling the sex difference in fat mass by adding weight in males reduced by 31,4% and 71.7% for height and power results (Fig. 2 and 3).

Due to the action of sex hormones, gradual increase in body fat is observed in girls during maturation [9]. Hanjabam and Meitei [43], reported that the differences in anthropometric parameters are due to the sex-specific post-pubescent hormonal status. They reported a positive correlation between jump performances and body lean mass and the skeletal mass component. Furthermore, Hanjabam et al. [41] demonstrated that height, body mass and back strength performance are the predictors of SJ height.

In this line, our results showed that male students are 15,7% heavier (*p* < 0.001), 7,4% taller (*p* < 0.001) and 44.0% (*p* < 0.001) stronger in back strength (BS) than female students.

According to Boisseau [12], because their weaker muscular volume and their higher percentage of body fat, women physical performance can never match that of their male counterparts. In this context, Rogol et al. [44] reported that hormone production during puberty causes changes in body composition, including changes in the relative proportions of water, muscle, fat and bone. In general, boys have a significant increase in bone and muscle growth and a simultaneous loss of fat in the limbs under the influence of testosterone. In agreement with the literature, we have found that male subjects are higher, heavier and less fat than female subjects.

The ballasting of male students was accompanied by an average of 1.5 ± 0.2 m reduction of the 5JT performance (i.e. a decrease of 13.3% in performance, Fig. 1). The sex differences (∆ F/wM in %) were also reduced to 11.7% (*p* < 0.001). The increased carried weight by male students led to an increase in the amount of energy required to perform the jumps. These data indicate that being overweight or under fat excess impair physical performance [45]. Our results are in agreement with those of Katralli and Goudar [46] who reported that the higher the percentage of fat, the lower the performance during activities involving movement of the body. Excess weight or body fat affects performance by lowering the energy available to move each kilogram of body weight [45]. In this context, Shephard [14] reported that body composition constitutes a factor disadvantaging female athletic performance since a woman must propel a greater fat mass with less muscle mass.

Our results show that when the average percentage of fat (i.e. an inactive tissue in the force-producing system) in men is made comparable to that in women, the difference in average performance between men and women is attenuated but does not disappeared. Thus, although body fat has a substantial effect on 5JT performance, it cannot be proposed as the sole or the main factor responsible for the differences in performance observed between the sexes. Other factors may contribute to this effect. Kacem et al. [26] hypothesized that volume muscle of the lower limbs which is more important in men can be considered as the main factor responsible for the differences in performance observed between the two sexes during 5JT.

In this line, weighting of male subjects caused a decrease in sex differences of SJ performance from 34.7% to 23.8% (height; *p* < 0.001) and from 24.9% to 7.0% (power; *p* < 0.001) Cancelling the sex difference in fat mass by adding weight in male students reduced by 71.7% the sex difference in the power outcome during SJ (Fig. 2 and 3). Our results corroborate those of Davis et al. [47] who reported that the percentage of body fat in athletes was negatively correlated with jump height. In addition, these authors reported that the percentage of body fat was the best predictor of vertical jump for men aged from 20 to 37 years practicing a leisure activity. This result agrees with those of Roschel et al. [48], who stipulated that the sum of the thicknesses of skin folds is negatively correlated with the performance of vertical jumping. Since work is the product of the average force acting on the subject and the displacement of the jump, heavier athletes need more work to bring the body to the same vertical displacement as that performed by lighter athletes [49].

In our study, although reduced, the sex differences persist significantly. This supposes that factors other than the fat mass in excess contributed to the difference in performance between male and female students. Thus, other biological (i.e. morphological, neuromuscular) and/or social (level of sport participation, practiced sports, training habits) factors might be major contributors for the noted differences in strength and power performance between male and female students.

## Conclusion

The performances of manual and back strengths, and power outcome during jumps are significantly better in male students compared to female ones. After ballasting the male students, the sex differences persist significantly. Concerning the power outcome during SJ, the sex difference in fat mass might account for almost 71% of the sex difference in power. We suggest that other factors such as muscle volume, hormonal differences, character, agility and other intrinsic factors may contribute to the differences observed between sexes.

In male or female subjects, excess of body fat is deleterious for strength and power performances. Hence, to approach or equalize male physical performance, women must begin as a first step by reducing their percentage of fat mass.

## Data Availability

The datasets used and/or analyzed during the current study available from the corresponding author on reasonable request.

## Abbreviations

Anova: Analysis of variance
BMI: Body mass index
BS: Back strength
∆ F/M: Differences between female and male
HG: Hand gripping
5JT: Five jump test
SJ: Squat jump
∆ F/wM: Differences between female and weighted male
